# Botulinum Toxin as an Adjunct to Bilateral Medial Rectus Recessions for Large Angle of Esotropia in High Myopic Patients: A Case Report

**DOI:** 10.7759/cureus.42580

**Published:** 2023-07-27

**Authors:** Saif Al-Obaisi, Taghreed Al-Najjar

**Affiliations:** 1 Pediatric Ophthalmology Department, King Abdullah Specialist Children Hospital, Riyadh, SAU

**Keywords:** strabismus surgery, botulinum toxin, bilateral medial rectus recession, high myopia, esotropia

## Abstract

Botulinum toxin injection adjunct to bilateral medial rectus recession is a documented procedure for correcting the large angle of infantile esotropia. A 9-year-old boy presented with a large angle of esotropia (80 PD) and high myopia. He underwent bilateral medial rectus recession with an adjunct botulinum toxin injection. Six months after the procedure, the patient had an esotropia of < 10 PD. Maximum bilateral medial rectus recession (BMR) with augmented botulinum toxin injection can be an effective procedure for large-angle acquired esotropia. This is helpful in avoiding an initial three-muscle operation.

## Introduction

Botulinum toxin started to be used as an alternative to weakening strabismus procedures, and it proved to have a long-term effect on the muscle [1]. It has benefits in young children or syndromic patients when measuring the angle cannot be accurate [2]. In some studies, it was approved to have an effect on small and large angles in the treatment of partial accommodative esotropia, but bilateral medial rectus recession (BMR) remains a standard of care [3]. Some surgeons start to use botulinum toxin as an adjunct to some procedures to maximize the corrected angle of deviation [4]. Using botulinum toxin as an adjunct therapy to the main surgery also has the benefit of reducing the number of muscles that need to be operated on [5]. In this report, we describe the use of botulinum toxin as an adjunct to maximum BMR for the treatment of large esotropia.

## Case presentation

**Figure 1:**
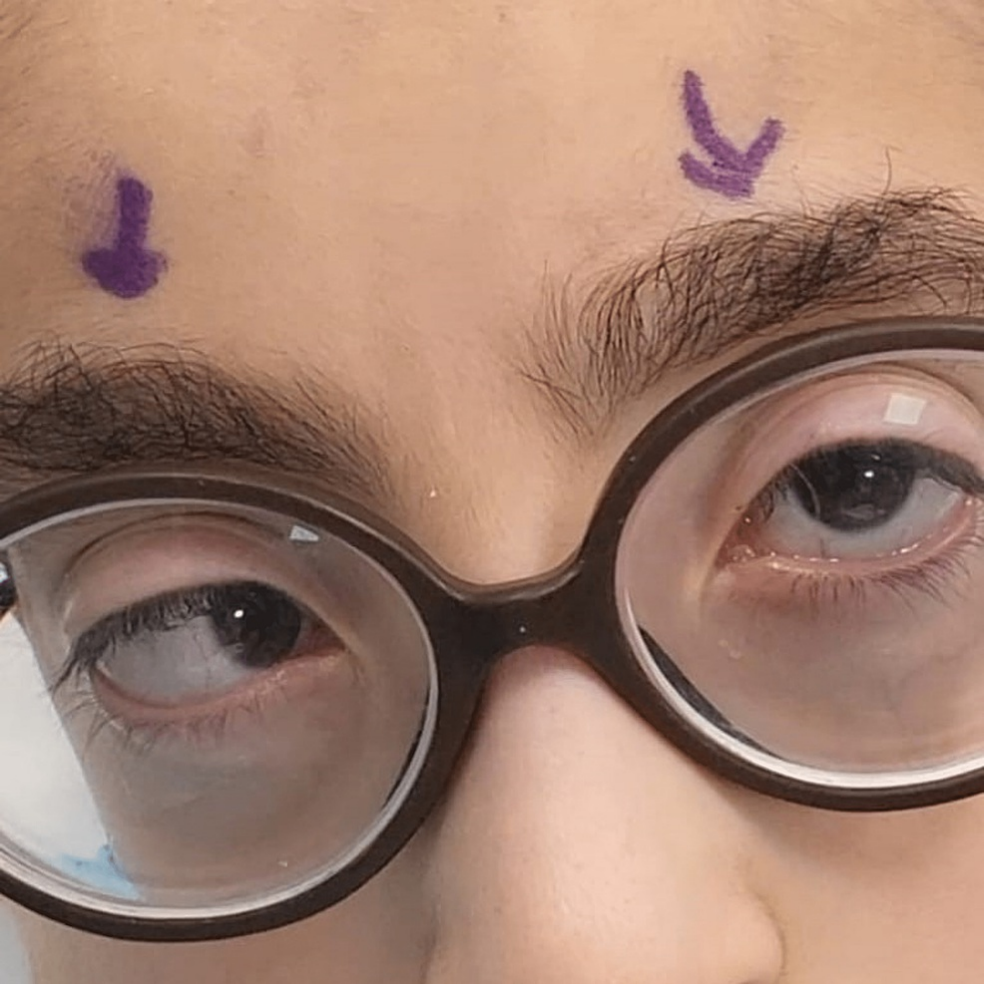
Large angle of esotropia before surgery.

A 9-year-old boy with a known case of myopia wore his glasses regularly; his parents complained of inward deviation with and without glasses and no diplopia. Upon examination, the vision in both eyes was 0.2 with correction. The refractive error in the right eye was -9.00-2.25x63 and in the left eye was -8.00-2.25x127. The patient wore full correction lenses. The extraocular movement was full, with no inferior oblique overaction. His orthoptic assessment with an alternate prism cover test for near and far with and without correction esotropia was 80 prismatic diopters (PD) with no pattern (Figure 1).

**Figure 2:**
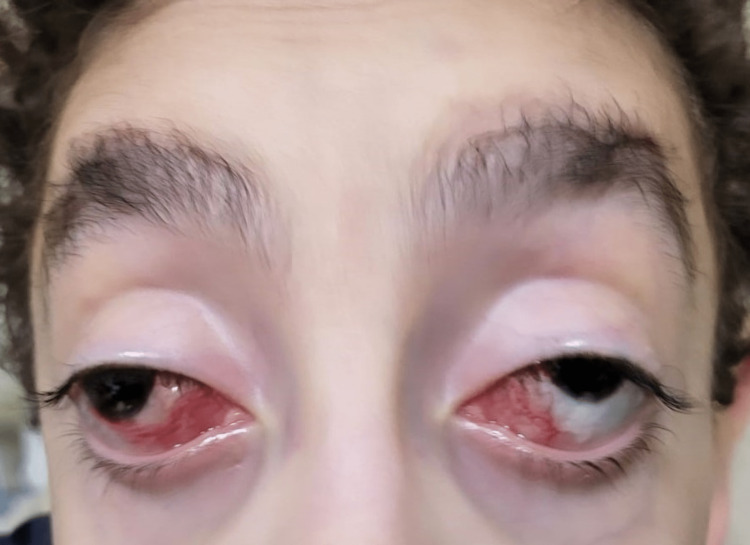
Large exotropia with ptosis three weeks post procedure

His anterior segment and posterior pole examinations were unremarkable, aside from his myopic fundus appearance. His cycloplegic refraction was almost the same as that while wearing glasses. Since he had a known case of high myopia with a large angle of esotropia, he underwent a large bilateral medial rectus recession with botulinum toxin injection in the same session after explaining all the benefits and risks to his parents, who signed the surgical consent form. A force duction test was performed before surgery, and there were no restrictions. We used the fornix approach, hooked the medial rectus, and isolated it. All the tenons and connective tissues were removed. A double-armed 6-0 Vicryl suture was used to suture the muscle with a locked knot at each end. The muscle was then disinserted from its insertion and attached to the globe at 7 mm posterior to the original insertion. Botulinum toxin (5 IU) was injected with direct visualization into the medial rectus, and the conjunctiva was closed using an 8-0 Vicryl suture. The first postoperative visit was three weeks after the procedure. His orthoptic assessment with an alternate prism cover test for near and far without correction was 50 PD exotropia with no pattern and marked ptosis in both eyes. Extraocular movement showed a limitation of adduction around -2 in both eyes (Figure 2).

**Figure 3:**
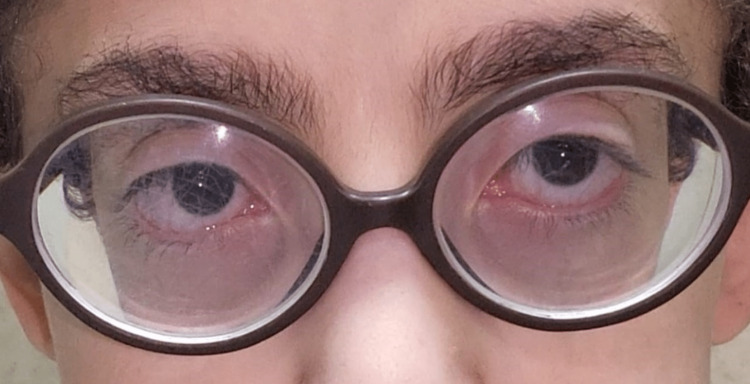
Almost ortho six months post-surgery

One month later, his exotropia was reduced to 25 PD near and far, with correction and no ptosis. Six-month post-procedure, he ended with a small angle of esotropia for near and far with a correction of less than 10 PD with an alternate prism cover test (Figure 3).

## Discussion

The patient had high myopia and a large angle of esotropia (50 PD). We administered an adjunct botulinum toxin injection with maximum BMR to achieve maximum correction of the deviation. This procedure is used in infantile esotropia with a large angle (mean, 72 PD), with a success rate of 72% at a mean follow-up of 6.6 years. This indicates the long-term impact of these procedures [6]. Wan et al. conclude their study that augmented botulinum toxin injection surgery can be used as an alternative to traditional surgery in the large angle of esotropia [7].

In addition, using botulinum toxin injections in conventional surgery will enhance the results by increasing the amount of expected correction [8]. By adding or augmenting the BMR with botulinum toxin injection, we can avoid working in three muscles by using this benefit. Using botulinum toxin injection with recession and resection for horizontal strabismus surgery showed this tendency [9]. The same result was reported in a previous study (n = 13) on the treatment of sensory deviation with monocular recession and resection with augmented botulinum toxin injection. The preoperative angle of deviation was above 50 PD, and the postoperative angle of deviation was less than 10 PD, with a mean follow-up period of 52.77 +/- 10.9 months [4].

Owens et al. did recession and resection for the large angle exotropia with botulinum toxin injection in three patients, and he also discussed the benefits of this technique to avoid the operation on three muscles [10]. In contrast, Mattout et al. compare the three-muscle surgery with the bilateral lateral rectus recession with augmented botulinum toxin injection [5]. They found that three muscle surgeries had a higher success rate than two muscle surgeries with augmented botulinum toxin injection, with success rates of 66.7% and 47.6%, respectively [5].

Heavy eye syndrome is esotropia in highly myopic patients secondary to inferior displacement of the lateral rectus and nasal displacement of the superior rectus with protrusion of the eye. In such situations, traditional recession and resection surgery may be ineffective. Loop myopexy with or without medial rectus recession will correct this pathway [11]. Multiple patients can present with high myopia without displacement of the extraocular muscle [12]. This patient presented with large-angle esotropia and high myopia as incidental findings that did not match the criteria for heavy-eye syndrome.

Regarding complications, this patient has neither persistent ptosis nor exotropia, both documented with botulinum toxin injection and conventional surgery [3].

## Conclusions

There are multiple approaches to correcting a large angle of deviation. Working on three muscles in one or multiple sessions can give good alignment. Botulinum toxin injections as adjuncts to conventional surgery can be alternating options. Maximum BMR with augmented botulinum toxin injection can be an effective procedure for large-angle acquired esotropia. This is helpful in avoiding an initial three-muscle operation.
